# Supplementary material for: Chemical Composition, Repellent Action, and Toxicity of Essential Oils from Lippia origanoide, Lippia. alba Chemotypes, and Pogostemon cablin on Adults of Ulomoides dermestoides (Coleoptera: Tenebrionidae)
Source: Insects. 2022 Dec 31;14(1):41. doi: 10.3390/insects14010041 (PMC9863295; doi:10.3390/insects14010041)
Supplement: Supplementary file 1 [file insects-14-00041-s001.zip › insects-2003370-supplementary.pdf]

SUPPLEMENTARY INFORMATION

Chemical composition, repellent action and toxicity of essential oils from *Lippia origanoide, L. alba* chemotypes and *Pogostemon cablin* on adults of *Ulomoides dermestoides* (Coleoptera: Tenebrionidae)

Caballero-Gallardo, Karina[1,2]., Fuentes-Lopez, Katerin[1,2]., Stashenko, Elena E[3]., Olivero-Verbel, Jesus[1*]

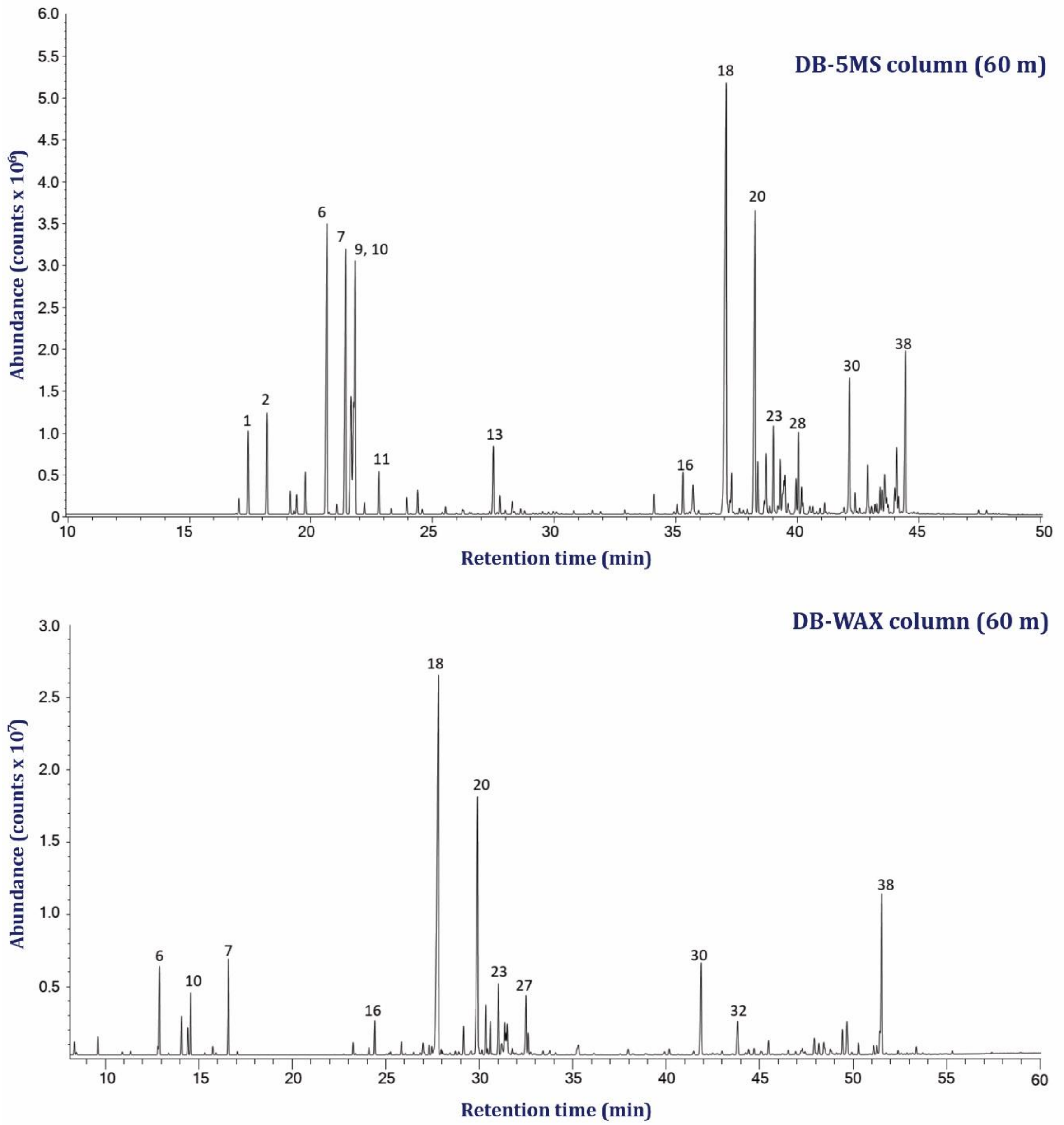

Figure S1. Chromatographic profiles obtained by GC/MS (full scan) of *L. origanoides*, phellandrene chemotype EO, DB-5MS and DB-WAX columns (60 m), *split* 1:30, MSD (EI, 70 eV). Compounds: α-Pinene (1), Camphene (2), Sabinene (3), β-Pinene (4), β-Myrcene (5), α-Phellandrene (6), *p*-Cymene (7), Limonene (8), β-Phellandrene (9), 1,8-Cineole (10), γ-Terpinene (11), Linalool (12), Borneol (13), Terpinen-4-ol (14), α-Cubebene (15), α-Copaene (16), β-Elemene (17), trans-β-Caryophyllene (18), β-Copaene (19), α-Humulene (20), γ-Muurolene (21), Amorpha-4,7(11)-diene (22), Germacrene D (23), β-Selinene (24), α-Muurolene (25), α-Selinene (26), δ-Cadinene (27), *cis*-Calamenene (28), N.I. M+• *m/z* 204 (**Figure S1a**) (29), Caryophyllene oxide (30), Guaiol (31), Humulene epoxide II (32), γ-Eudesmol (33), Coelution N.I. M+•

m/*z* 220 + N.I. M+• m/z 204 (34), Caryophylla-4(12),8(13)-dien-5β-ol (35), *α*-Cadinol (36), *α*-Eudesmol (37), and N.I. M+• *m*/*z* 222 (**Figure S1b**) (38).

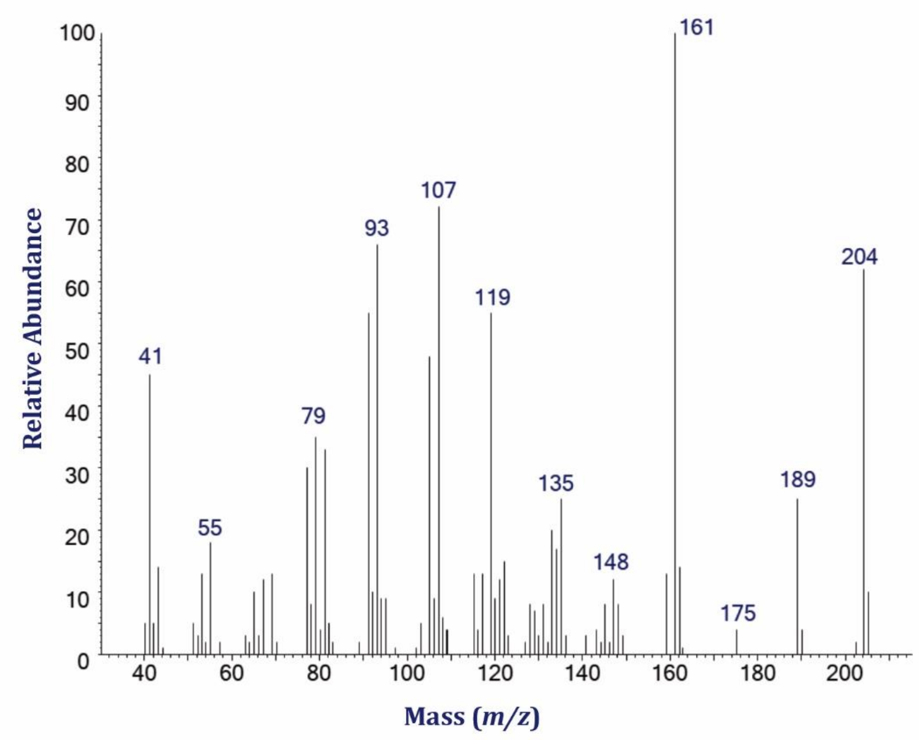

Figure S1a. Mass spectra (EI, 70 eV) of the unidentified compound, Peak No. 29 (N.I. M+• *m*/*z* 204).

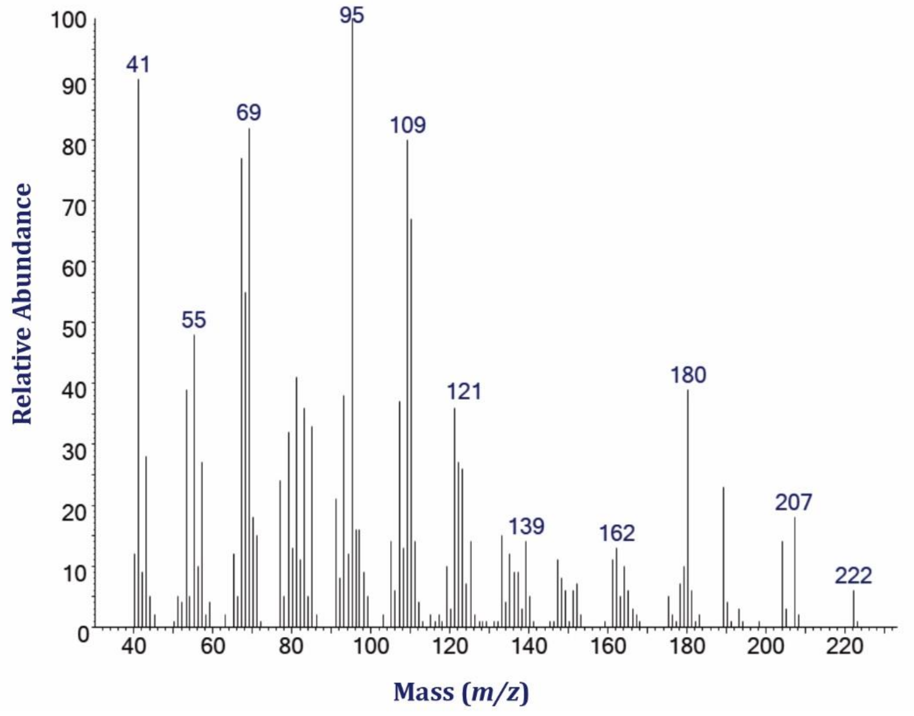

Figure S1b. Mass spectra (EI, 70 eV) of the unidentified compound, Peak No. 38 (N.I. M+• *m/z* 222).

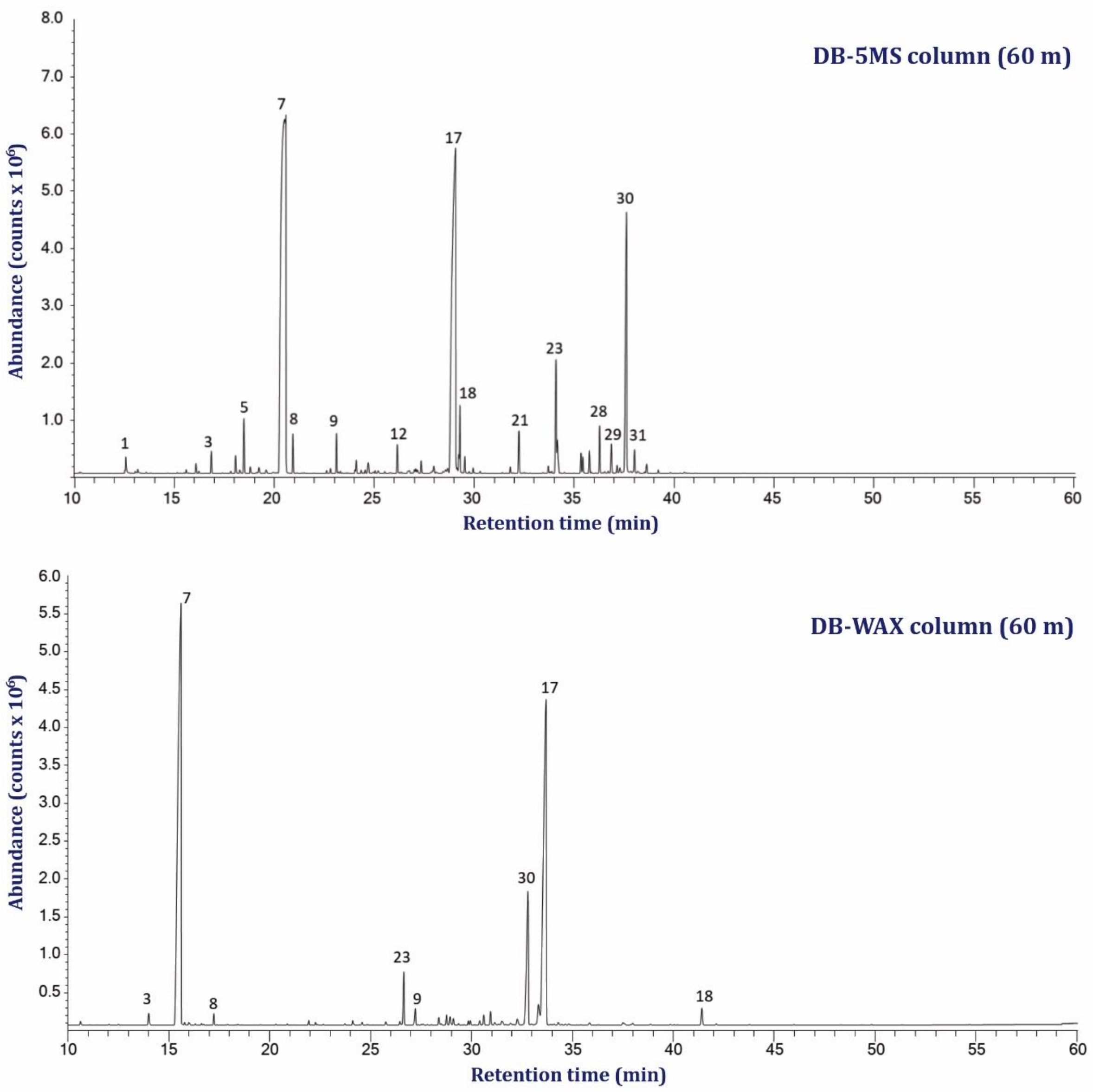

Figure S2. Chromatographic profiles obtained by GC/MS (full scan) of *L. alba*, carvone chemotype EO, DB-5MS and DB-WAX columns (60 m), *split* 1:30, MSD (EI, 70 eV). Compounds: *cis*-Hex-3-en-1-ol (1), α-Pinene (2), Camphene (3), Oct-1-en-3-ol (4), β-Mircene (5), Octan-3-ol (6), Limonene (7), *trans*-β-Ocimene (8), Linalool (9), *trans*-*p*-Mentha-2,8-dien-1-ol (10), *cis*-Limonene oxide (11), Borneol (12), *cis*-Dihydrocarvone (13), *trans*-Dihydrocarvone (14), *cis*-Carveol (15), neo-iso-Dihydrocarveol (16), Carvone (17), Piperitone (18), Geranial (19), *trans*-Carvyl acetate (20), Piperitenone (21), α-Copaene (22), β-Bourbonene (23), β-Elemene (24), β-Copaene (25), *trans*-β-Caryophyllene (26), β-Gurjunene (27), *trans*-β-Farnesene

(28), *trans*-9-epi-Caryophyllene (29), Germacrene D (30), Bicyclogermacrene (31), and α-Cadinene (32).

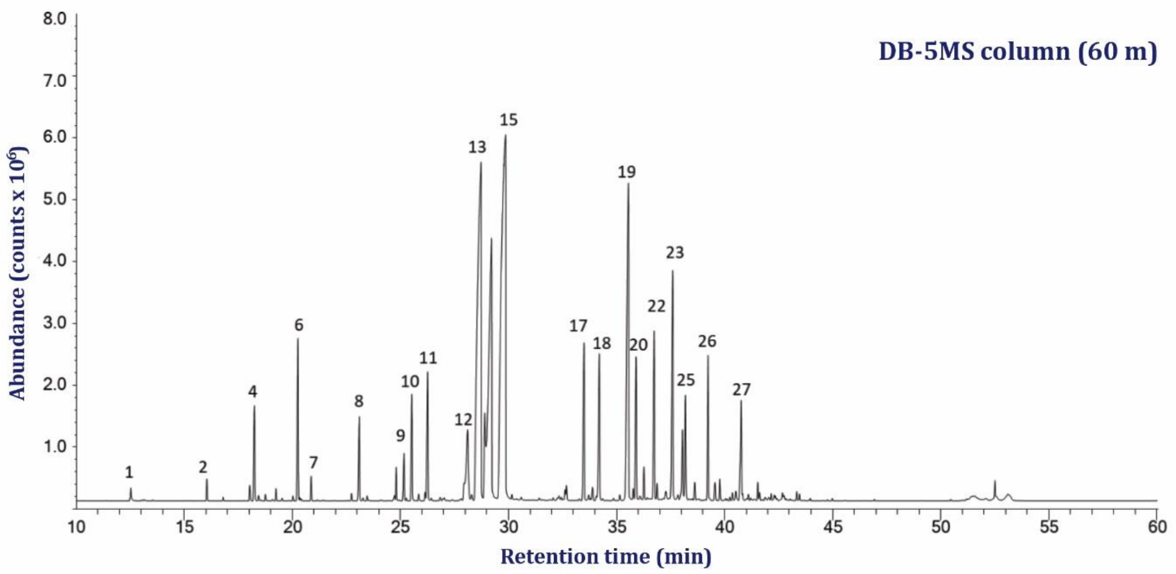

Figure S3. Chromatographic profiles obtained by GC/MS (full scan) of *L. alba*, citral chemotype EO, DB-5MS column (60 m), *split* 1:30, MSD (EI, 70 eV). Compounds: *cis*-Hex-3-en-1-ol (1), α-Pinene (2), Oct-1-en-3-ol (3), 6-Methyl-hept-5-en-2-ona (4), α-Phellandrene (5), Limonene (6), *trans*-β-Ocimene (7), Linalool (8), Citronellal (9), Isocitral (10), Isogeranial (11), Nerol (12), Neral (13), Geraniol (14), Geranial (15), Neryl acetate (16), β-Elemene (17), Geranyl acetate (18), *trans*-β-Caryophyllene (19), *trans*-β-Caryophyllene (20), *trans*-β-Farnesene (21), α-Humulene (22), Germacrene D (23), Geranyl isobutanoate (24), α-Bulnesene (25), *trans*-α-Bisabolene (26), Caryophyllene oxide (27), N.I. M+• m/z 286 (**Figure S3a**) (28).

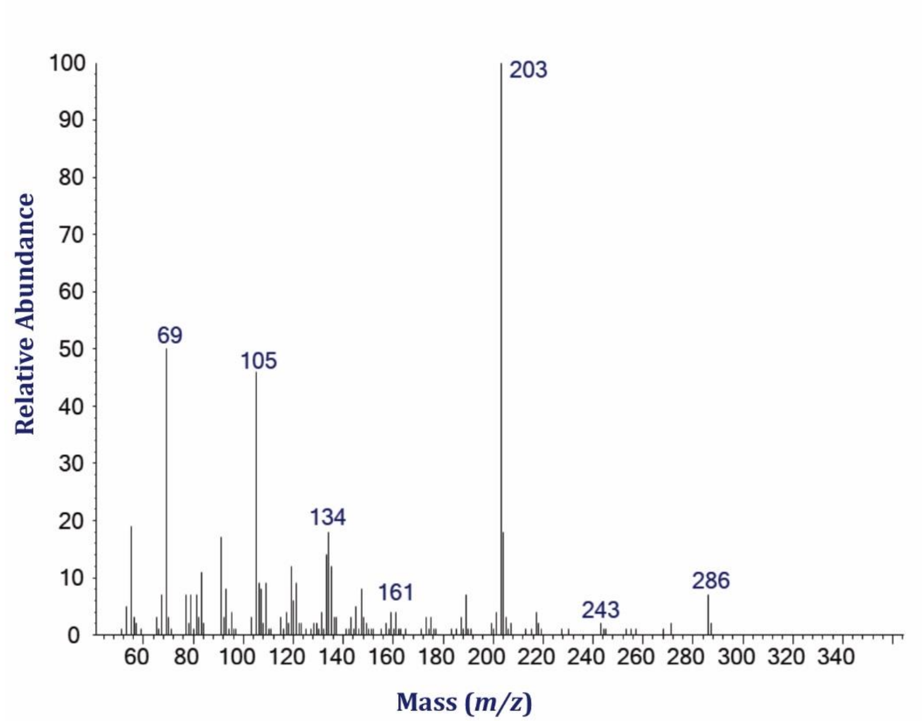

Figure S3a. mass spectra (EI, 70 eV) of the unidentified compound, Peak No. 286 (N.I. M+• m/z 286).

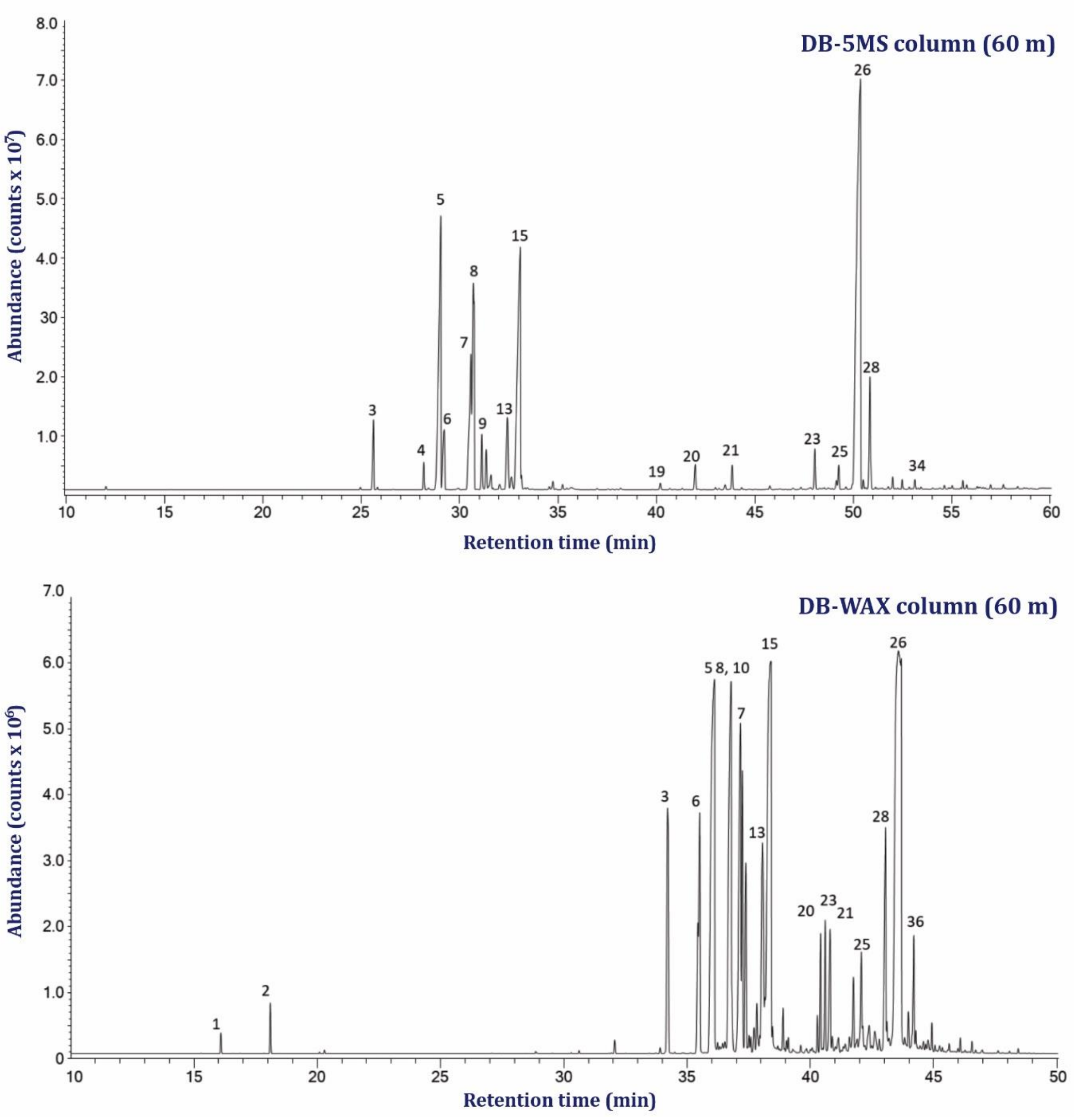

Figure S4. Chromatographic profiles obtained by GC/MS (full scan) of *P. cablin* EO, DB-5MS and DB-WAX columns (60 m), *split* 1:30, MSD (EI, 70 eV). Compounds: α-Pinene (1), β-Pinene (2), β-Patchoulene (3), Cycloseychellene (4), α-Patchoulene (5), *trans*-β-Caryophyllene (6), α-Patchoulene (7), Seychellene (8), γ-Patchoulene (9), α-Humulene (10), N.I. $M^{+\bullet}$ *m/z* 204 (**Figure S4a**) (11), δ-Selinene (12), Aciphyllene (13), γ-Gurjunene (14), α-Bulnesene (15), α-Selinene (16), 7-epi-α-Selinene (17), Nootkatene (18), N.I. $M^{+\bullet}$ *m/z* 220 (**Figure S4b**) (19), N.I. $M^{+\bullet}$ *m/z* 220 (**Figure S4c**) (20), Caryophyllene oxide (21), Humulene epoxide II (22), Norpatchoulenol (23), N.I. $M^{+\bullet}$ *m/z* 222 (**Figure S4d**) (24), N.I. $M^{+\bullet}$ *m/z* 222 (**Figure S4e**) (25), Patchoulol (26), N.I. $M^{+\bullet}$ *m/z* 206 (**Figure S4f**) (27), Pogostol (28), Coelution N.I. M+• m/z 222 + N.I. M+• m/z 220 (29), Rotundone (30), N.I. $M^{+\bullet}$ *m/z* 220 (**Figure S4g**) (31), N.I. $M^{+\bullet}$ *m/z* 222 (**Figure S4h**) (32), N.I. $M^{+\bullet}$ *m/z* 218

(**Figure S4i**) (33), Dehydrofukinone (34), N.I. $M^{+\bullet}$ *m*/*z* 218 (**Figure S4j**) (35), and Pogostone (36).

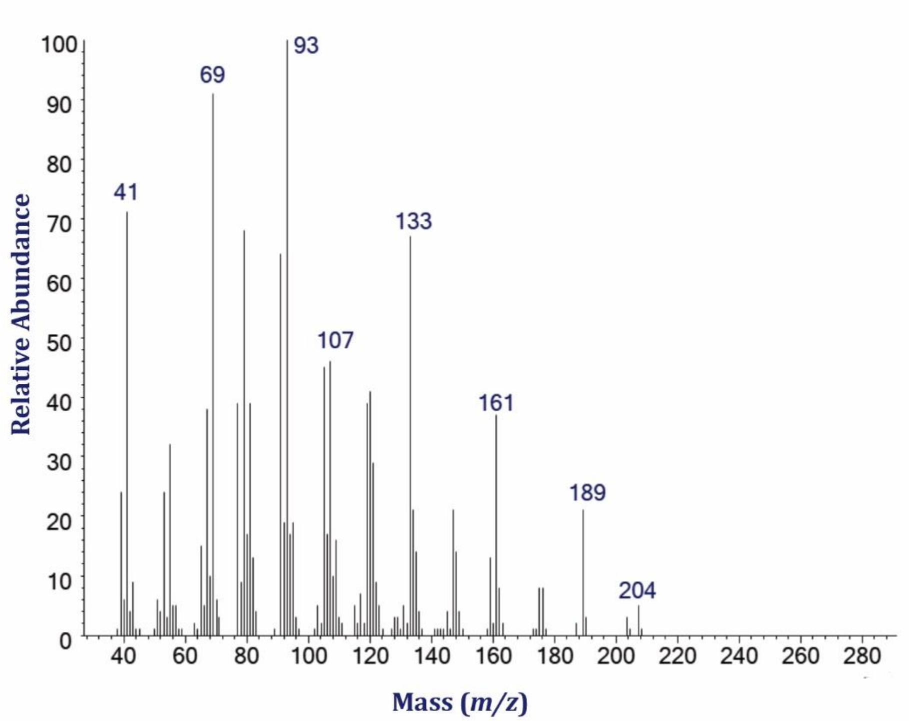

Figure S4a. Mass spectra (EI, 70 eV) of the unidentified compound, Peak No. 204 (N.I. $M^{+\bullet}$ *m*/*z* 204).

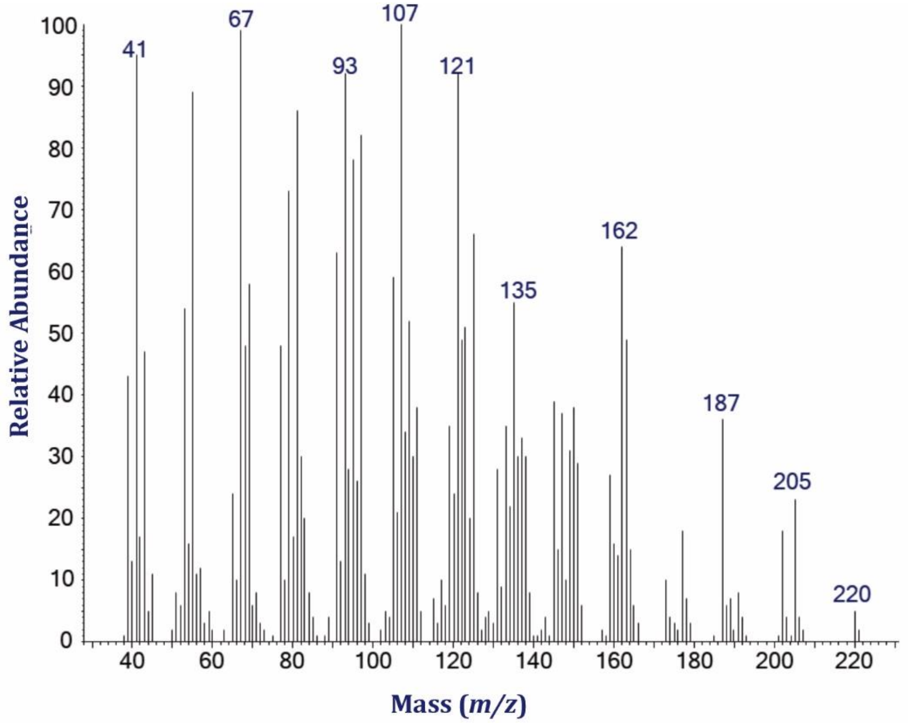

Figure S4b. Mass spectra (EI, 70 eV) of the unidentified compound, Peak No. 220 (N.I. $M^{+\bullet}$ *m*/*z* 220).

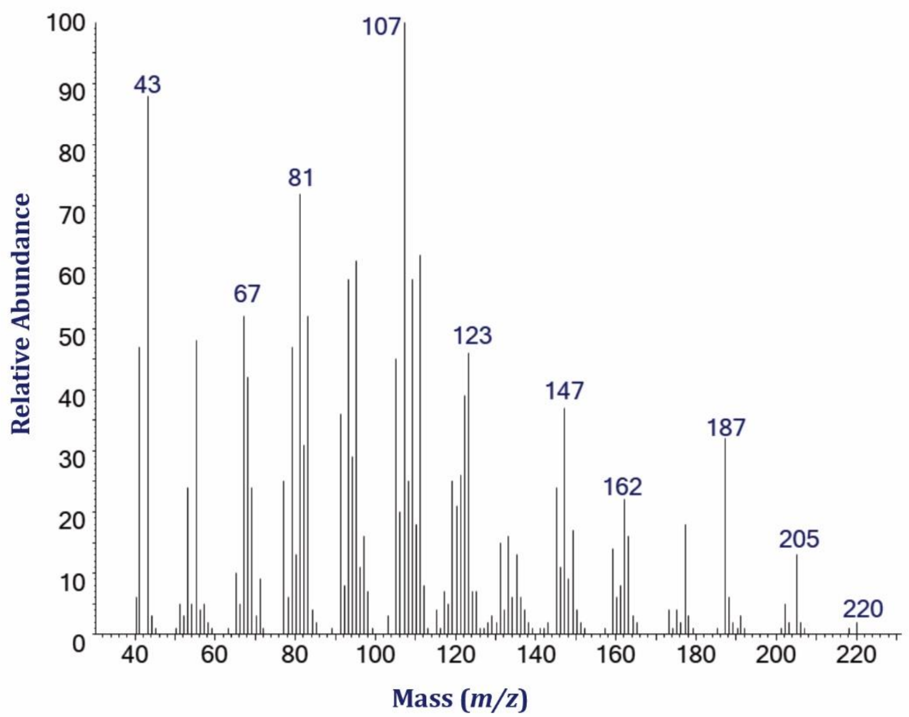

Figure S4c. Mass spectra (EI, 70 eV) of the unidentified compound, Peak No. 220 (N.I. $M^{+\bullet}$ *m*/*z* 220).

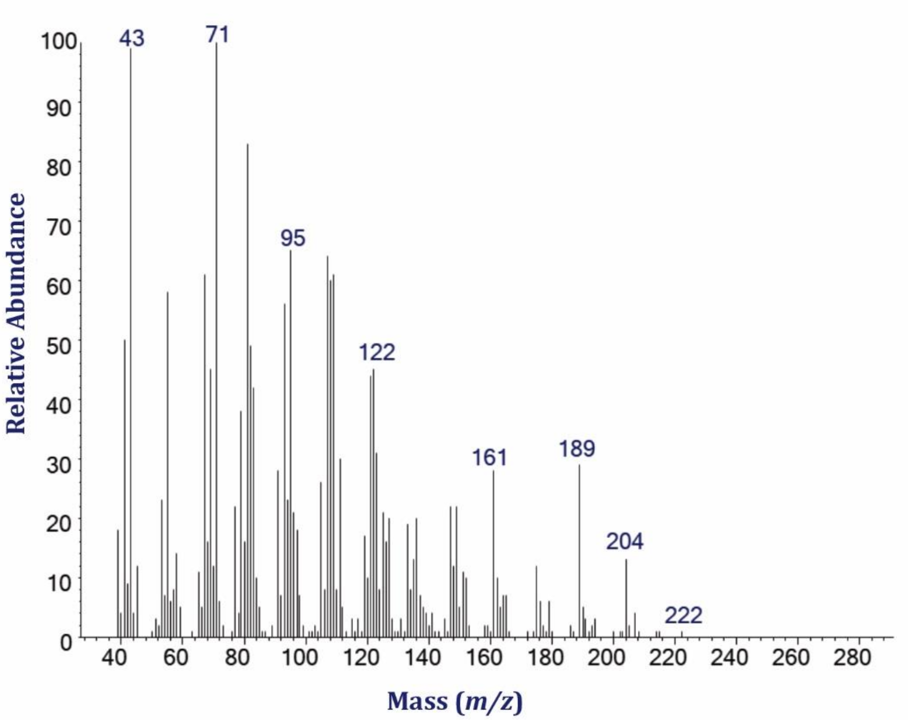

Figure S4d. Mass spectra (EI, 70 eV) of the unidentified compound, Peak No. 222 (N.I. $M^{+\bullet}$ *m*/*z* 222).

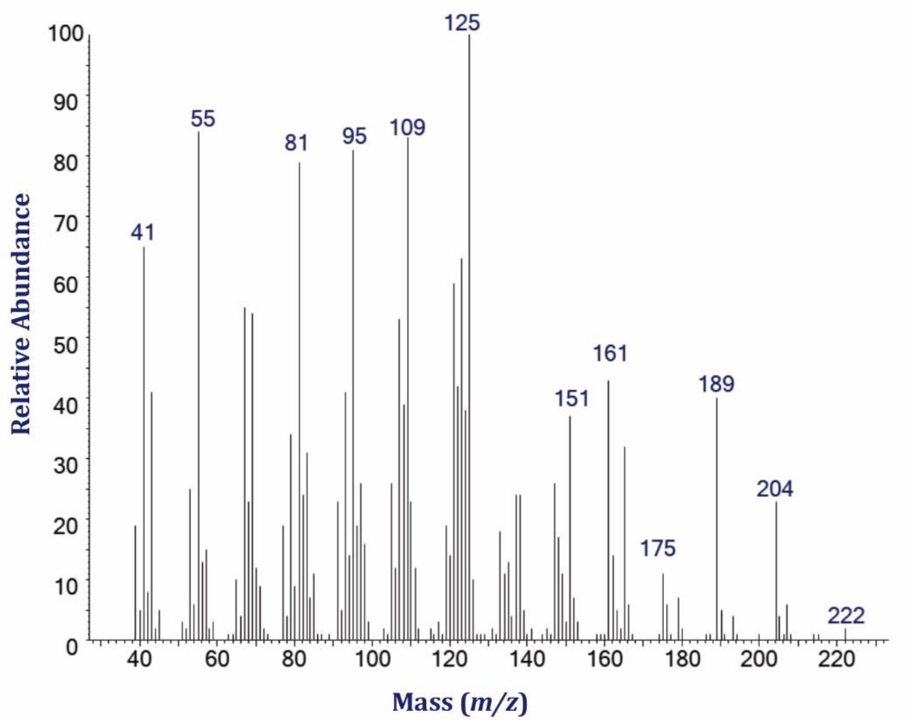

Figure S4e. Mass spectra (EI, 70 eV) of the unidentified compound, Peak No. 222 (N.I. $M^{+\bullet}$ *m/z* 222).

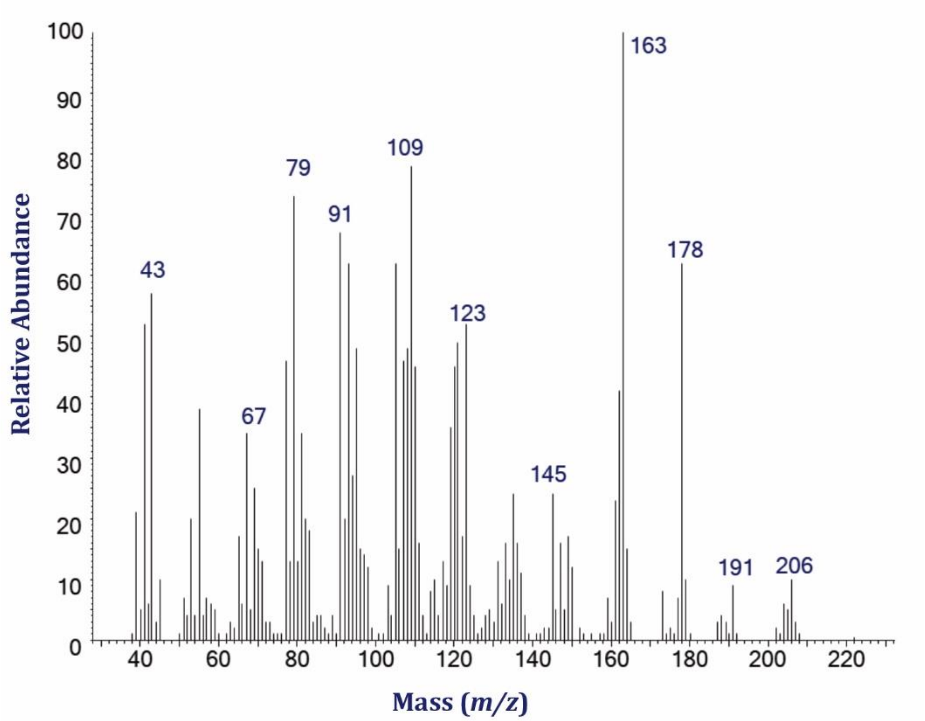

Supplementary Figure S4f. Mass spectra (EI, 70 eV) of the unidentified compound, Peak No. 206 (N.I. $M^{+\bullet}$ *m/z* 206).

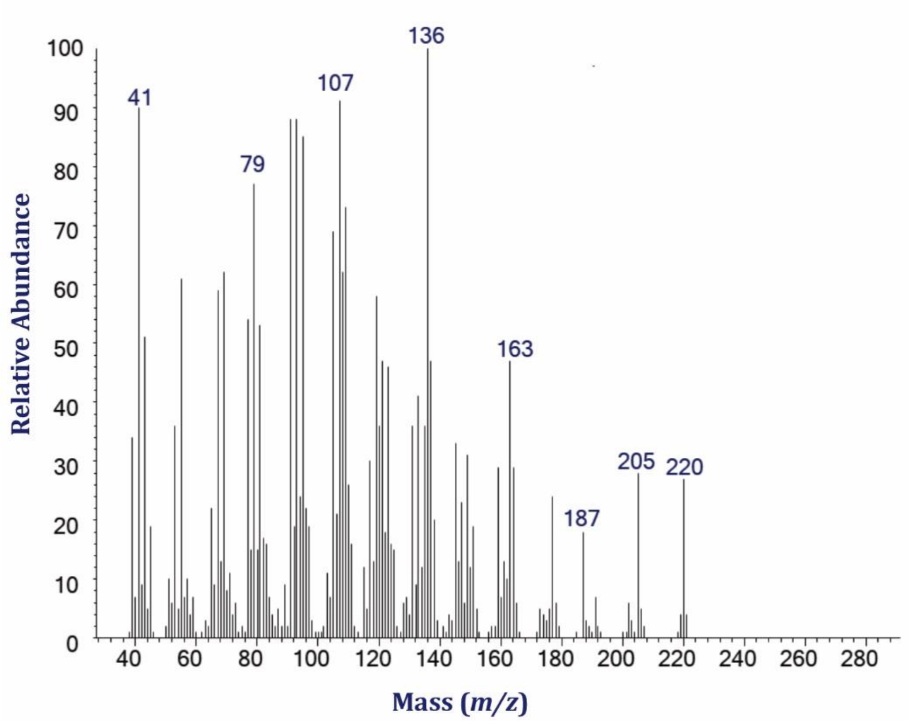

Figure S4g. Mass spectra (EI, 70 eV) of the unidentified compound, Peak No. 220 (N.I. $M^{+\bullet}$ *m*/*z* 220).

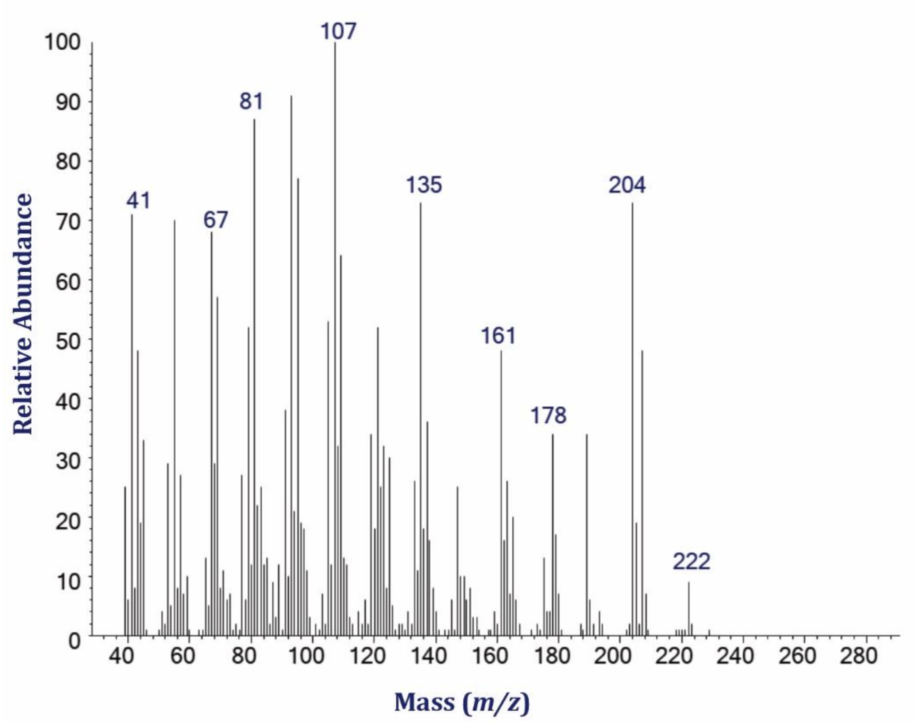

Figure S4h. Mass spectra (EI, 70 eV) of the unidentified compound, Peak No. 222 (N.I. $M^{+\bullet}$ *m*/*z* 222).

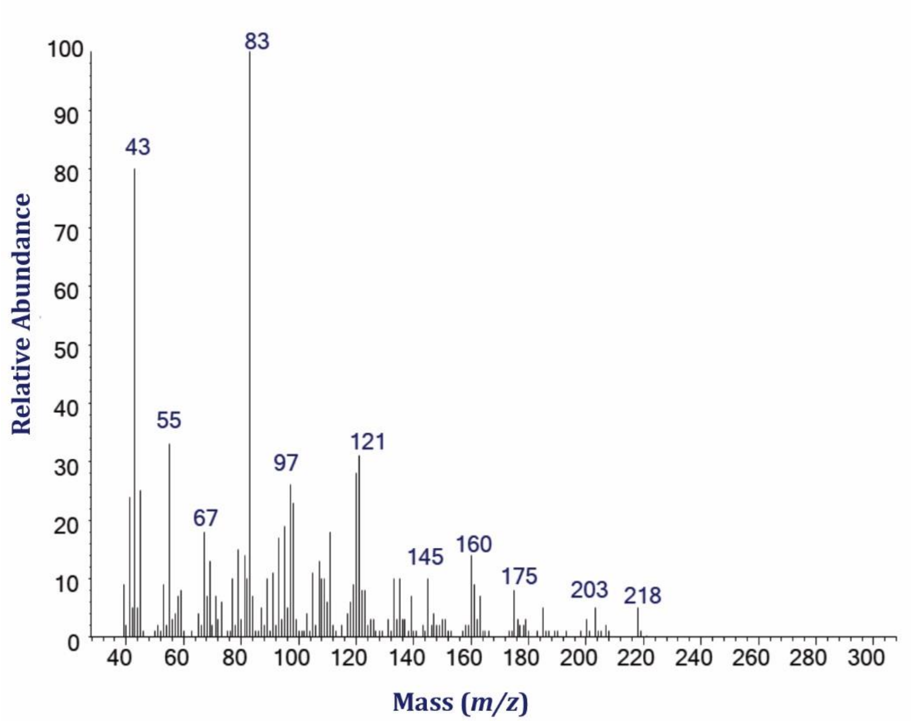

Figure S4i. Mass spectra (EI, 70 eV) of the unidentified compound, Peak No. 218 (N.I. $M^{+\bullet}$ *m/z* 218).

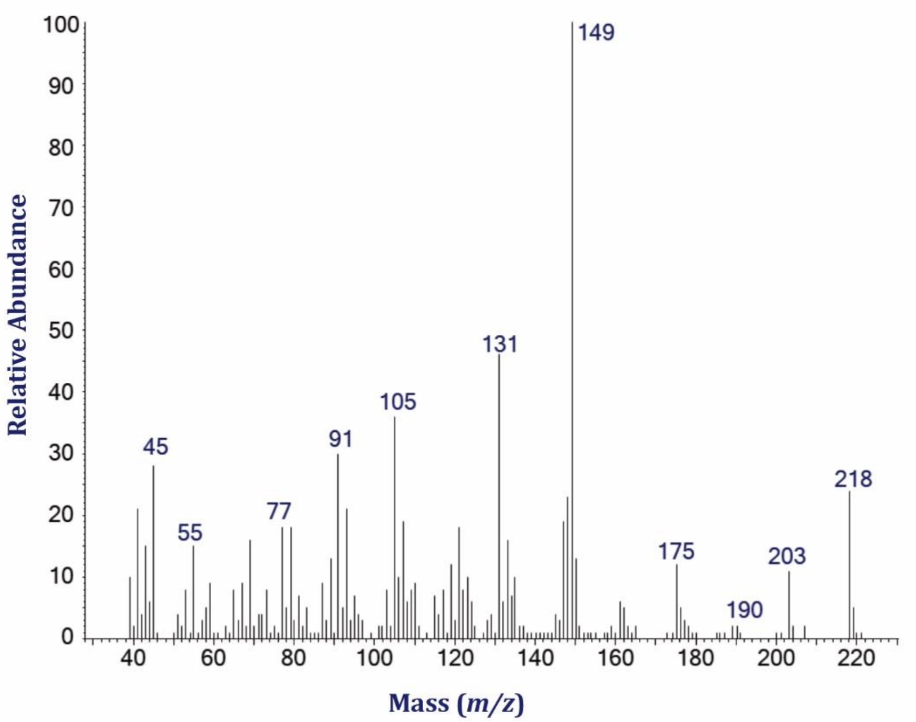

Figure S4j. Mass spectra (EI, 70 eV) of the unidentified compound, Peak No. 218 (N.I. $M^{+\bullet}$ *m/z* 218).
